# Six-month survival and quality of life of intensive care patients with acute kidney injury

**DOI:** 10.1186/cc13076

**Published:** 2013-10-22

**Authors:** Sara Nisula, Suvi T Vaara, Kirsi-Maija Kaukonen, Matti Reinikainen, Simo-Pekka Koivisto, Outi Inkinen, Meri Poukkanen, Pekka Tiainen, Ville Pettilä, Anna-Maija Korhonen

**Affiliations:** 1 Intensive Care Units, Division of Anaesthesia and Intensive Care Medicine, Department of Surgery, Helsinki University Central Hospital, Haartmaninkatu 4 00029, Helsinki, Finland; 2 Australian and New Zealand Intensive Care Research Centre, Department of Epidemiology and Preventive Medicine, Monash University, Melbourne, Australia; 3 Department of Intensive Care, North Karelia Central Hospital, Joensuu, Finland; 4 Department of Anesthesia and Intensive Care, Vaasa Central Hospital, Vaasa, Finland; 5 Department of Anesthesia and Intensive Care Medicine, Turku University Hospital, Turku, Finland; 6 Department of Anesthesia and Intensive Care Medicine, Lapland Central Hospital, Rovaniemi, Finland; 7 Department of Intensive Care, South Karelia Central Hospital, Lappeenranta, Finland; 8 Institute of Clinical Medicine, University of Helsinki, Helsinki, Finland

## Abstract

**Introduction:**

Acute kidney injury (AKI) has high incidence among the critically ill and associates with dismal outcome. Not only the long-term survival, but also the quality of life (QOL) of patients with AKI is relevant due to substantial burden of care regarding these patients. We aimed to study the long-term outcome and QOL of patients with AKI treated in intensive care units.

**Methods:**

We conducted a predefined six-month follow-up of adult intensive care unit (ICU) patients from the prospective, observational, multi-centre FINNAKI study. We evaluated the QOL of survivors with the EuroQol (EQ-5D) questionnaire. We included all participating sites with at least 70% rate of QOL measurements in the analysis.

**Results:**

Of the 1,568 study patients, 635 (40.5%, 95% confidence interval (CI) 38.0-43.0%) had AKI according to the Kidney Disease Improving Global Outcomes (KDIGO) criteria. Of the 635 AKI patients, 224 (35.3%), as compared to 154/933 (16.5%) patients without AKI, died within six months. Of the 1,190 survivors, 959 (80.6%) answered the EQ-5D questionnaire at six months. The QOL (median with Interquartile range, IQR) measured with the EQ-5D index and compared to age- and sex-matched general population was: 0.676 (0.520-1.00) versus 0.826 (0.812-0.859) for AKI patients, and 0.690 (0.533-1.00) versus 0.845 (0.812-0.882) for patients without AKI (*P* <0.001 in both). The EQ-5D at the time of ICU admission was available for 774 (80.7%) of the six-month respondents. We detected a mean increase of 0.017 for non-AKI and of 0.024 for AKI patients in the EQ-5D index (*P* = 0.728). The EQ-5D visual analogue scores (median with IQR) of patients with AKI (70 (50–83)) and patients without AKI (75 (60–87)) were not different from the age- and sex-matched general population (69 (68–73) and 70 (68–77)).

**Conclusions:**

The health-related quality of life of patients with and without AKI was already lower on ICU admission than that of the age- and sex-matched general population, and did not change significantly during critical illness. Patients with and without AKI rate their subjective health to be as good as age and sex-matched general population despite statistically significantly lower QOL indexes measured by EQ-5D.

## Introduction

Acute kidney injury (AKI) has a high incidence of up to 30% to 40% [1-3] among the critically ill and is associated with a dismal outcome [3,4]. Almost 40% of patients suffering from severe AKI (KDIGO, Kidney Disease: Improving Global Outcomes, Stage 3) die within 90 days [3]. Furthermore, only 30% of patients receiving renal replacement therapy (RRT) due to AKI are alive five years after admission to the ICU [5]. In addition, AKI associates with permanently deteriorated kidney function and chronic dialysis dependency [6]. Despite initial functional recovery, patients treated with RRT remain at permanent risk of developing end-stage renal disease [7].

The majority of studies reporting the long-term survival and health-related quality of life (QOL) of kidney injury patients focus on RRT patients [5,8,9], but high mortality and an association with considerable morbidity is not isolated to the severe stages of AKI. Only a few long-term outcome and QOL studies focus on AKI and define AKI with any of the latest consensus criteria (KDIGO, Acute Kidney Injury Network (AKIN) or Risk, Injury, Failure, Loss of kidney function and End-stage kidney disease (RIFLE)). Most of these studies are retrospective or limited to a certain subgroup of patients [10-15].

Accordingly, we followed a heterogeneous group of critically ill patients who were included in the large, prospective observational multi-centre FINNAKI study [3]. In this predetermined sub-study we aimed to evaluate the six-month survival and QOL of these critically ill patients and explore factors associated with good QOL after AKI. We hypothesised that patients with AKI would have significantly lower QOL compared to those without AKI and also lower QOL than the age-and sex-matched general Finnish population.

## Materials and methods

### Patients

We performed a six-month follow-up of 2,901 ICU patients included in the prospective, multi-centre FINNAKI study during the period 1 September 2011 to 1 February 2012. In brief, the study included consecutive emergency ICU patients and elective (postoperative) patients, whose stay exceeded 24 hours. The FINNAKI study in detail has been published previously [3]. In this pre-determined follow-up study we aimed to measure QOL of all six-month survivors. We decided *a priori* to include ICUs with at least a 70% follow-up rate in the final analysis. Each patient or proxy gave written informed consent for the study. The Ethics Committee of the Department of Surgery in Helsinki University Hospital gave approval for the study.

### Definitions and data collection of FINNAKI

We measured creatinine (Cr) daily and urine output hourly and defined AKI with the KDIGO guidelines [16]. If baseline creatinine (latest value from the previous year excluding the previous week) was not available, we used the Modification in Diet in Renal Disease (MDRD) equation assuming a glomerular filtration rate of 75 ml/1.73 m^2^[17]. We collected patient demographics, medical history, severity scores, length-of stay, physiologic data, and the EuroQol (EQ-5D) questionnaire responses from the Finnish Intensive Care Consortium prospective database (Tieto Ltd, Helsinki, Finland) and with a study specific case report form. We obtained data on survival at six months from the Finnish Population Register Centre. We collected physiologic data and screened the patients for the presence of AKI and severe sepsis for five days starting from ICU admission.

### Health-related quality of life

The QOL of the included patients at ICU admission and at six months was measured with the EQ-5D [18] questionnaire. The EQ-5D is a standardized, multidimensional instrument found suitable for critically ill patients [19,20]. The EQ-5D includes five dimensions (mobility, self-care, usual activities, pain/discomfort, and anxiety/depression) evaluated on a scale of 1 to 3. Calculating a single index score (0 to 1) combines these five dimensions. This value can be used in comparisons between different populations. The questionnaire includes a visual analogue scale (VAS) (values from 0 to 100) for recording the respondent’s self-rated health. According to previous reports a significant change in the EQ-5D index is 0.08, and for the VAS score 7 [21,22]. The ICU nurse presented the EQ-5D questionnaire to the patient or proxy when collecting the admission data. Data obtained from proxies have been shown to be reliable [23,24]. At six months, the questionnaire was presented by mail or by telephone.

### Statistical analysis

We present continuous data as medians with interquartile ranges (IQR) and absolute values and percentages with 95% confidence intervals (CI). We used the two-tailed Mann–Whitney *U* test or the Kruskal-Wallis test for comparison of continuous variables and the chi-square test for categorical variables. We compared the EQ-5D index and reference values using the Wilcoxon signed matched pair test. The analysis for the change in EQ-5D indexes was performed as a paired samples analysis. We explored potential independent factors associated with good QOL (defined as at least equal to that of age- and sex matched controls) at six months. First, we tested plausible factors in univariable models and, second, we entered significant variables (*P* <0.20) into a multivariable model (logistic regression). Additional file 1 lists all the tested and inserted variables. We considered a *P* value less than 0.05 as significant unless stated otherwise. We performed all analyses by SPSS version 20 (SPSS, Chicago, Ill., USA).

## Results

### Patients

**Figure 1:**
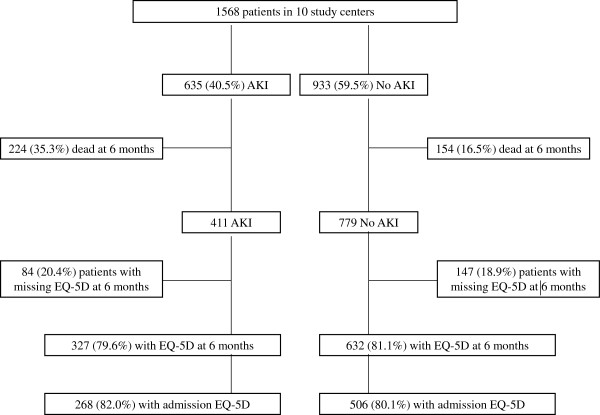
The study flow chart.

The study flow chart is presented in Figure 1. Altogether 1,568 patients from ten ICUs with at least a 70% QOL response rate were included in the final analysis. Characteristics of the study patients are presented in Table 1 and a comparison to the whole FINNAKI study cohort is in Additional file 2. The incidence of AKI (95% CI) in this substudy was 635/1,568 (40.5%, 38.0% to 43.0%). Of all patients (95% CI), 280 (17.9%, 15.9% to 19.8%) had stage 1, 119 (7.6%, 6.3 to 8.9%) had stage 2, and 236 (15.1%, 13.2% to 16.9%) had stage 3 AKI. During the first five ICU treatment days 162/1,568 (10.3%, 8.8% to 11.9%) patients received RRT.

**Table 1:** Patient characteristics of all study patients (N = 1,568) and patients with and without AKI

	**Data available (of 1,568)**	**All patients (N = 1,568)**	**No AKI patients (N = 933)**	**AKI patients (N = 635)**
		**N (%) or median (IQR)**	**N (%) or median (IQR)**	**N (%) or median (IQR)**
Age (years)	1,568	65 (53 to 74)	64 (50 to 73)	66 (56 to 76)
Gender (male)	1,568	1015 (64.7)	583 (62.5)	432 (68.0)
Baseline serum/plasma creatinine (μmol/l)	1,088	78 (63 to 95)	77 (61 to 93)	79 (65 to 101)
Co-morbidity				
Chronic obstructive pulmonary disease	1,560	169 (10.8)	91 (9.8)	78 (12.3)
Hypertension	1,567	759 (48.4)	411 (44.1)	348 (54.8)
Arteriosclerosis	1,554	236 (15.1)	116 (12.6)	120 (18.9)
Diabetes	1,568	359 (22.9)	200 (21.4)	159 (25.0)
Systolic heart failure	1,561	219 (14.0)	115 (12.3)	104 (16.5)
Chronic kidney disease	1,562	127 (8.1)	48 (5.1)	79 (12.4)
Admission type				
Emergency	1,540	1,287 (82.1)	751 (81.9)	536 (86.0)
Surgical	1,568	618 (39.4)	377 (40.4)	241 (38.0)
APACHE II Diagnostic group				
Cardiovascular, operative		355 (22.6)	208 (22.3)	147 (23.2)
Cardiovascular, non-operative		231 (14.7)	121 (13.0)	110 (17.3)
Respiratory tract, non-operative		184 (11.7)	115 (12.3)	69 (10.9)
Gastrointestinal tract, operative		152 (9.7)	86 (9.2)	66 (10.4)
Metabolic		133 (8.5)	93 (10.0)	40 (6.3)
Sepsis		99 (6.3)	43 (4.6)	56 (8.8)
Gastrointestinal tract, non-operative		92 (5.9)	48 (5.1)	44 (6.9)
Neurological, non-operative		85 (5.4)	69 (7.4)	16 (2.5)
Trauma		70 (4.5)	52 (5.6)	18 (2.8)
Other (<5% each)		167 (10.7)	98 (10.5)	69 (10.9)
Severity and outcome				
SOFA (first 24 hours, points)	1,568	7 (5 to 10)	6 (4 to 8)	9 (6 to 11)
SAPS II score (points)	1,568	36 (27 to 49)	32 (25 to 43)	42 (33 to 58)
Length of ICU stay (days)	1,568	2.8 (1.6 to 5.4)	2.1 (1.3 to 4.4)	3.8 (2.0 to 7.1)
Length of hospital stay (days)	1,566	9 (5 to 17)	8 (5 to 14)	11 (5 to 19)
Six-month mortality	1,568	378 (24.1)	154 (16.5)	224 (35.3)

APACHE II, Acute Physiology and Chronic Health Evaluation II; SAPS II, Simplified Acute Physiology Score; SOFA, Sequential Organ Failure Assessment.

### Six-month mortality

The overall six-month mortality (95% CI) was 378/1,568 (24.1%, 21.9% to 26.3%). Of the 635 AKI patients, 224 (35.3%, 95% CI 31.5% to 39.1%) died within six months, as compared to 154/933 (16.5%, 95% CI 14.1% to 18.9%) patients without AKI. The six-month mortality for patients with RRT was 63/162 (38.9%, 95% CI 31.2% to 46.5%).

### Health-related quality of life

Of the 1,190 patients who were alive at six months, 959 (80.6%) answered the EQ-5D questionnaire. The median response time was 232 days. The characteristics of the respondents and non-respondents at six months were comparable (data not shown).

Table 2 presents the distribution of the EQ-5D health questionnaire answers in different patient groups at six months. The EQ-5D index and VAS at six months after ICU admission for different patient groups and compared to the age- and sex-matched general population are presented in Table 3.

**Table 2:** EQ-5D (EuroQol) health dimensions at six months after ICU admission

	**All patients (N = 959) %**	**Patients without AKI (N = 632) %**	**Patients with AKI (N = 327) %**	**Patients with RRT (N = 85) %**	**Stage 3 patients without RRT (N = 29) %**
Mobility					
I have no problems in walking about	52.3	54.3	48.6	43.5	44.8
I have some problems in walking about	41.0	39.7	43.4	48.2	44.8
I am confined to bed	6.7	6.0	8.0	8.2	10.3
Self-care					
I have no problems with self-care	75.9	77.8	72.2	63.5	65.5
I have some problems washing or dressing myself	18.5	16.6	22.0	28.2	24.1
I am unable to wash or dress myself	5.6	5.5	5.8	8.2	10.3
Usual activities (for example, work, study, housework, family or leisure activities)					
I have no problems with performing my usual activities	57.7	59.2	54.7	57.6	55.2
I have some problems with performing my usual activities	33.2	32.4	34.6	31.8	31.0
I am unable to perform my usual activities	9.2	8.4	10.7	10.6	13.8
Pain/discomfort					
I have no pain or discomfort	46.4	47.9	43.4	40.0	48.3
I have moderate pain or discomfort	47.8	46.5	50.2	51.8	44.8
I have extreme pain or discomfort	5.8	5.5	6.4	8.2	6.9
Anxiety/depression					
I am not anxious or depressed	71.8	71.0	73.4	72.9	82.8
I am moderately anxious or depressed	25.3	26.3	23.5	25.9	13.8
I am extremely anxious or depressed	2.8	2.7	3.1	1.2	3.4

**Table 3:** The health-related quality of life by the EQ-5D (EuroQol) index and visual analogue scale (VAS) at six months after ICU admission compared to the age- and sex-matched general population

	**EQ-5D index**		**EQ-5D VAS**	
	**Study patients**	**General population**	**Study patients**	**General population**
All study patients (N = 959)	0.690 (0.533-1.00)^a^	0.845 (0.812-0.882)	73 (59–85)	70 (68–77)
No AKI patients (N = 632)	0.690 (0.533-1.00)^a^	0.845 (0.812-0.882)	75 (60–87)	70 (68–77)
AKI patients (N = 327)	0.676 (0.520-1.00)^a^	0.826 (0.812-0.859)	70 (50–83)	69 (68–73)
Stage 1 (N = 148)	0.691 (0.534-1.00)^a^	0.826 (0.812-0.876)	75 (60–86)	69 (68–76)
Stage 2 (N = 65)	0.631 (0.442-0.795)^a^	0.826 (0.774-0.859)	69 (50–82)	69 (65–73)
Stage 3 (N = 114)	0.676 (0.486-1.00)^a^	0.845 (0.826-0.859)	70 (50–80)	70 (68–73)
With RRT (of stage 3) (N = 85)	0.676 (0.482-0.802)^a^	0.845 (0.819-0.882)	65 (50–80)^a^	70 (68–77)
Without RRT (of stage 3) (N = 29)	0.676 (0.504-1.00)	0.826 (0.826-0.852)	70 (50–90)	68 (68–73)

^a^*P* <0.001, comparison between the study patients and the age- and sex-matched general population.

Values are presented as median (interquartile range, IQR). AKI, acute kidney injury (by the Kidney Disease Improving Global Outcomes, KDIGO criteria); RRT, renal replacement therapy.

**Figure 2:**
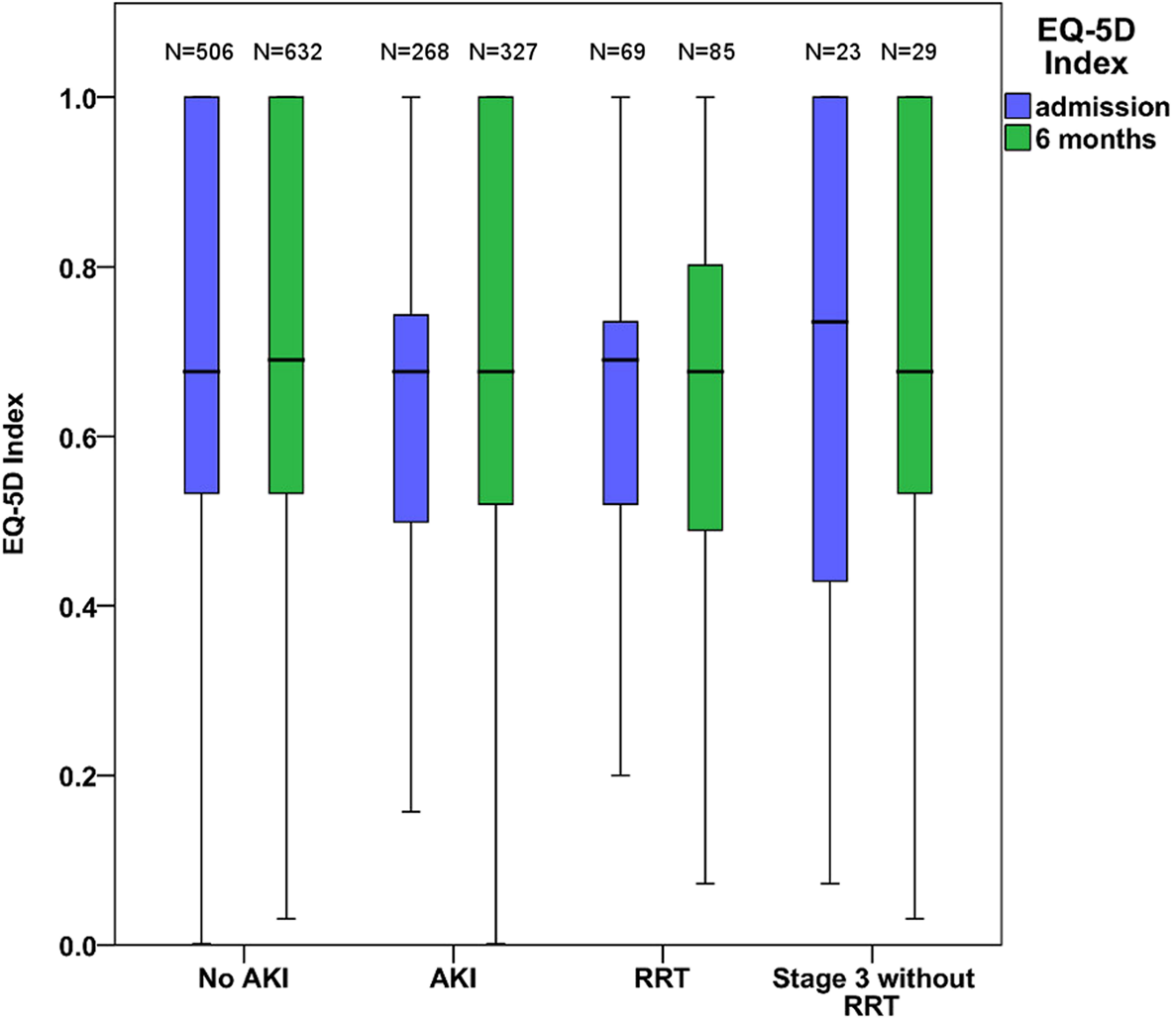
Boxplot of the changes in the EQ-5D index during six months from ICU admission stratified into different patients groups.

Of the six-month respondents (N = 959), the admission EQ-5D was available for 774 (80.7%). Of these, 615 (79.4%) were answered by the patient, 139 (18.0%) by a proxy, and in 20 (2.6%) the respondent was unknown. In these 774 patients the mean increases in the EQ-5D index during the six-month follow-up were 0.017 (no AKI) and 0.024 (AKI). The mean difference (95% CI) between the mean changes for non-AKI and AKI patients was 0.007 (-0.314 to 0.045) (*P* = 0.728). The changes in the EQ-5D index within six months following ICU admission in different patients groups are illustrated in Figure 2.

Of 1,568 study patients, 390 (24.9%) did not respond to the EQ-5D questionnaire on ICU admission. These non-respondents on ICU admission had higher severity scores compared to the respondents (day 1 SOFA score (IQR) 8 (6 to 10), as compared to 7 (5 to 9), and SAPS II score (IQR) 40 (31 to 55), as compared to 35 (26 to 47)).

Of the 378 deceased patients, 223 (59.0%) had responded to the EQ-5D questionnaire at ICU admission. The median (IQR) admission EQ-5D index of patients who died within six months from admission was significantly lower (*P* <0.001) compared to the admission EQ-5D of those who survived (0.533 (0.356 to 0.690) versus 0.676 (0.520 to 0788)).

Of the 959 six-month respondents, 311 (32.4%) achieved a good QOL defined as EQ-5D index which was equal or superior to that of age- and sex matched general population. Of the 327 AKI patients, 96 (29.4%) achieved this good QOL. When exploring potential predictors of good QOL after AKI, we found that (1) the EQ-5D score at admission (odds ratio (OR) (95% CI) 1.042 (1.024 to 1.060)/0.01 points), and (2) lack of hypertension (OR 2.561 (1.141 to 5.750) were independent predictors of a good QOL six months after ICU admission. In non-AKI patients only the admission EQ-5D index (OR 1.039 (1.028 to 1.049)/0.01 points) was an independent predictor of good QOL at six months. The variables tested in univariable models and variables inserted into the multivariable models are listed in Additional file 1. In this study population a longer hospital length-of stay (LOS) was associated with lower QOL (*P* <0.001) [see Additional file 3].

## Discussion

In this large prospective, multi-centre observational study in Finnish ICUs we found that two-thirds of the AKI patients were alive at six months after ICU admission. The health-related QOL of AKI patients was lower than that of the age- and sex-matched general population, but equal to critically ill patients without AKI.

### Six-month mortality

We reported a six-month mortality of 35.3% for AKI patients, and 16.5% for patients without AKI. The only previous, prospective study found six-month mortality of AKI patients (defined by RIFLE, AKIN or KDIGO criteria) to be 46.5% [13]. In addition, two retrospective, single-center studies have reported six-month mortality rates of 58.5% [10] and 38.0% [25] for AKI patients.

The long-term outcome of RRT patients has been more extensively studied [26]. In our study the six-month mortality for RRT patients was 38.9%. Three other ICU studies, all retrospective, have reported higher six-month mortality rates for RRT patients: 49.4% [27], 59.9% [28], and 74.6% [29].

### Health-related quality of life

The QOL of survivors measured six months after ICU admission did not differ between patients who had developed AKI and those who had not. Moreover, the QOL did not change during this follow-up period for either group. However, the QOL of ICU patients was already significantly lower than that of the age-and sex-matched general population at the time of ICU admission and remained so during the follow-up period (Table 3). Recently, comparable results obtained with a different QOL questionnaire were reported by Hofhuis *et al*. [13]. In contrast, in another study from 2009, AKI patients had a lower QOL six months after surgery compared to patients without AKI [25].

Other QOL studies in AKI patients have only included RRT patients, and all have reported impaired health compared to controls [5,27,30-34], in concordance with our results. A randomised, controlled trial comparing different intensities of RRT reported low QOL scores for RRT patients at 60 days [6]. Compared to our study, however, that study had a different QOL measure (Health Utilities Index (HUI) versus EQ-5D), follow-up time (60 days versus 180 days), patient population (only RRT versus all AKI patients) and reported no admission QOL data or VAS scores. Two Finnish studies with the same QOL questionnaire used in our study (EQ-5D) also reported that the QOL of RRT patients was lower compared to the matched general population [5,27].

RRT patients, despite their impaired QOL, have self-rated their quality of life as equal to that of the age- and sex-matched general population [5,27] or stated that they would choose to undergo the same treatment again [30,32]. It has been suggested that surviving critical illness affects how people value their life and they are, therefore, content with health, which by objective measures is lower than that of the general population. In our study, AKI patients were as content with their QOL as the general population, and ICU patients without AKI evaluated their QOL to be even better compared to the general population by VAS. RRT patients, however, reported significantly lower VAS scores compared to the general population (Table 3).

The QOL of AKI and non-AKI patients was significantly lower than that of the general population already at the time of ICU admission which is in concordance with previous data [35]. In addition, the change in QOL in survivors of critical illness was minimal. This indicates that critical illness per se may not impact the QOL, but patients who get critically ill have existing poor health. Furthermore, patients who died during critical illness had a significantly lower EQ-5D at ICU admission compared to patients who survived.

One third of the survivors and one third of the AKI patients had an equal or superior QOL compared to the age- and sex-matched general population at six months. We found only two independent predictors of good QOL at six months after AKI: a higher EQ-5D index at ICU admission was associated with a higher probability of a good QOL and lack of hypertension as a chronic condition added the probability of a good QOL. Surprisingly, age, chronic conditions other than hypertension, or events prior to ICU admission were not associated with the QOL outcome at six months in AKI patients. For comparison, in non-AKI patients only the EQ-5D index at admission was an independent predictor of a good QOL at six months in this study population. According to a systematic review from 2005, older age and increasing severity of illness may be associated with poorer outcome in some QOL dimensions in EQ-5D (physical function and general health perception) [35]. In our population, the patients with the highest tertile of LOS had the lowest QOL at six months in agreement with one previous study [6]. The systematic review from 2005, however, found no association between ICU LOS and QOL [35]. Furthermore, hospital LOS is a non-normally distributed factor that is also affected by factors unrelated to the patient. These findings reflect the difficulties in finding individual markers that would help us predict which patients will have a favorable outcome in the form of a good QOL after ICU treatment.

### Limitations and strengths

Our study has some limitations. First, although the mortality data at six months were anticipated to be complete due to the national registry, we expected that QOL data would be incomplete in some sites. Thus, to provide a reliable analysis we decided to include only those sites with at least a 70% QOL response rate. This decision resulted in the exclusion of 7 of 17 sites. Second, only 80% of those patients who survived in the included ten sites answered the EQ-5D questionnaire at six months. Finally, the pre-admission QOL was not available for 19% of six-month respondents. Thus, our analysis of QOL change between these two time-points was not complete. However, of the patients without AKI, one could assume that those patients who responded were slightly less ill than those who did not respond. In fact, that was the case and, thus, our finding of a non-significant difference of mean changes of QOL in critically ill patients with AKI and without AKI seems to be more reliable.

An obvious strength of our study is the prospective, multi-centre design and consecutive inclusion of a large number of patients both with and without AKI in the same ICUs and the availability of admission QOL data. In addition, we aimed to explore carefully the inevitable selection bias commonly seen in QOL studies in the critical care setting.

## Conclusions

We conclude that two thirds of critically ill AKI patients survive up to six months after ICU admission. Contrasting with our hypothesis, the six-month QOL of surviving AKI patients was comparable to that of surviving critically ill patients without AKI. The QOL of ICU patients was already lower at the time of ICU admission than that of the age- and sex-matched general population and was preserved in surviving patients. However, the perceived QOL of six-month-survivors was comparable to that of the age- and sex-matched general population with the exception of RRT patients.

## Key messages

• Two thirds of critically ill AKI patients survived up to six months after ICU admission.

• The six-month health-related quality of life of surviving AKI patients was comparable to that of surviving critically ill patients without AKI.

• The health-related quality of life of patients with and without AKI was already lower on ICU admission than that of the age- and sex-matched general population and did not change significantly during critical illness.

• AKI patients rated their subjective health to be as good as the age and sex-matched general population despite statistically significantly lower indexes measured by EQ-5D.

## Abbreviations

AKI: Acute kidney injury; AKIN: Acute Kidney Injury Network; CI: Confidence interval; Cr: Creatinine (serum or plasma); EQ-5D: EuroQol questionnaire; IQR: Interquartile range; KDIGO: Kidney Disease, Improving Global Outcomes; LOS: Length of stay; MDRD: Modification of diet in renal disease; QOL: Quality of life; RIFLE: Risk, Injury, Failure, Loss of kidney function and End-stage kidney disease; RRT: Renal replacement therapy; SAPS II: Simplified Acute Physiology Score; SOFA: Sequential Organ Failure Assessment; VAS: Visual analogue scale.

## Competing interests

The authors declare that they have no competing interests.

## Authors’ contributions

SN drafted the manuscript, performed the statistical analyses, and participated in the design and data gathering of the study. STV participated in the design and data gathering of the study and helped to draft the manuscript and to perform the statistical analyses. KMK participated in the design and coordination of the study, and revised the manuscript. MR participated in the design and data gathering of the study, and revised the manuscript. SPK, OI, MP and PT participated in the design and data gathering of the study, and revised the manuscript. VP participated in the design and coordination of the study and helped to draft the manuscript and to perform the statistical analyses. AMK participated in the design and coordination of the study, and helped to draft the manuscript. The FINNAKI-QOL study group members all participated in the local study coordination and data gathering. All authors read and approved the manuscript.

## Supplementary Material

Additional file 1 Variables tested in univariable models and variables inserted into the multivariable models for predicting a good quality of life (equal or superior to age- and sex-matched controls) in patients with and without acute kidney injury. Click here for file

Additional file 2 Comparison of characteristics of patients in this study and patients in the FINNAKI study. Click here for file

Additional file 3 EQ-5D index at six months stratified into groups based on hospital length-of stay. Click here for file

## The FINNAKI-QOL Study Group

*Central Finland Central Hospital:* Raili Laru-Sompa, Anni Pulkkinen, Minna Saarelainen, Mikko Reilama, Sinikka Tolmunen, Ulla Rantalainen, Marja Miettinen. *Helsinki University Central Hospital:* Ville Pettilä, Kirsi-Maija Kaukonen, Anna-Maija Korhonen, Sara Nisula, Suvi Vaara, Raili Suojaranta-Ylinen, Leena Mildh, Mikko Haapio, Laura Nurminen, Sari Sutinen, Leena Pettilä, Helinä Laitinen, Heidi Syrjä, Kirsi Henttonen, Elina Lappi, Hillevi Boman. *Jorvi Central Hospital:* Tero Varpula, Päivi Porkka, Mirka Sivula Mira Rahkonen, Anne Tsurkka, Taina Nieminen, Niina Prittinen. *Lapland Central Hospital:* Meri Poukkanen, Esa Lintula, Sirpa Suominen *Middle Ostrobothnia Central Hospital:* Tadeusz Kaminski, Fiia Gäddnäs, Tuija Kuusela, Jane Roiko. *North Karelia Central Hospital:* Sari Karlsson, Matti Reinikainen, Tero Surakka, Helena Jyrkönen, Tanja Eiserbeck, Jaana Kallinen. *South Karelia Central Hospital:* Seppo Hovilehto, Anne Kirsi, Pekka Tiainen, Tuija Myllärinen, Pirjo Leino, Anne Toropainen. *Turku University Hospital:* Outi Inkinen, Niina Koivuviita, Jutta Kotamäki, Anu Laine *Vaasa Central Hospital:* Simo-Pekka Koivisto, Raku Hautamäki, Maria Skinnar.
