# Supplementary material for: Six-month survival and quality of life of intensive care patients with acute kidney injury
Source: Crit Care. 2013 Oct 22;17(5):R250. doi: 10.1186/cc13076 (PMC4056803; doi:10.1186/cc13076)
Supplement: Additional file 1 — Variables tested in univariable models and variables inserted into the multivariable models for predicting a good quality of life (equal or superior to age- and sex-matched controls) in patients with and without acute kidney injury. [file cc13076-S1.doc]

**Additional file 1**. Variables tested in univaribale models and variables inserted into the multivariable models for predicting a good quality of life (equal or superior to age- and sex-matched controls) in patients with and without acute kidney injury.

| AKI | No AKI |
| --- | --- |
| Variables tested in univariate models | |
| 1. Admission EQ-5D index 2. Gender 3. Age 4. Statin medication 5. Metformin medication 6. Aspirin medication 7. Diuretics medication 8. Chorticosteroid medication 9. Chronic kidney disease 10. Diabetes 11. Systolic heart failure 12. Atherosclerosis 13. Hypertension 14. Chronic obsructive pulmonary disease 15. Pre-ICU hypovolemia 16. Pre-ICU rhabdomyolysis 17. Pre-ICU cardiogenic shock 18. Pre-ICU CPR 19. Pre-ICU hypotensio 20. Pre-ICU diuretics 21. Pre-ICU radiocontrast dye 22. Pre-ICU albumin 23. Pre-ICU HES 24. Operative admission 25. Non-emergency admission 26. Emergency surgery (<1 week) 27. Highest lactate during first 24 hours 28. SOFA score day 1 29. SAPSII score 30. Baseline creatinine 31. Renal replacement therapy 32. Severe sepsis 33. NYHA | 1. Admission EQ-5D index 2. Gender 3. Age 4. Statin medication 5. Metformin medication 6. Aspirin medication 7. Diuretics medication 8. Chorticosteroid medication 9. Chronic kidney disease 10. Diabetes 11. Systolic heart failure 12. Atherosclerosis 13. Hypertension 14. Chronic obsructive pulmonary disease 15. Pre-ICU hypovolemia 16. Pre-ICU rhabdomyolysis 17. Pre-ICU cardiogenic shock 18. Pre-ICUCPR 19. Pre-ICU hypotensio 20. Pre-ICU diuretics 21. Pre-ICU radiocontrast dye 22. Pre-ICU albumin 23. Pre-ICU HES 24. Operative admission 25. Non-emergency admission 26. Emergency surgery (<1 week) 27. Highest lactate during first 24 hours 28. SOFA score day 1 29. SAPSII score 30. Baseline creatinine 31. Severe sepsis 32. NYHA |
| Variables inserted into multivariable models | |
| 1. Admission EQ-5D index 2. Age 3. Statin medication 4. Aspirin medication 5. Diuretics medication 6. Chorticosteroid medication 7. Diabetes 8. Systolic heart failure 9. Atherosclerosis 10. Hypertension 11. Chronic obsructive pulmonary disease 12. Pre-ICU CPR 13. Pre-ICU hypotensio 14. Pre-ICU diuretics 15. Operative admission 16. Non-emergency admission 17. Highest lactate during first 24 hours 18. SOFA score day 1 19. SAPSII score 20. Renal replacement therapy 21. Severe sepsis 22. NYHA | 1. Admission EQ-5D index 2. Age 3. Diuretics medication 4. Chorticosteroid medication 5. Chronic kidney disease 6. Diabetes 7. Atherosclerosis 8. Hypertension 9. Chronic obsructive pulmonary disease 10. Pre-ICU hypotensio 11. Pre-ICU hypovolemia 12. Pre-ICU albumin 13. Operative admission 14. Non-emergency admission 15. Highest lactate during first 24 hours 16. SOFA score day 1 17. SAPSII score 18. Severe Sepsis |

Hypovolemia, by clinicans´ judgement

Rhabdomyolysis, CK > 5000 U/l or myoglobin > 5000 µg/l

CPR, hemodynamic collapse requiring chest compressions, defibrillation or administration of adrenalin

Hypotension, systolic blood pressure < 90 mmHg for 1 hour

HES, hydroxyethyl starch

SOFA, Sequential Organ Failure Assessment

SAPS II, Simplified Acute Physiology Score

NYHA, The New York Heart Association Functional Classification
