# Supplementary material for: Six-month survival and quality of life of intensive care patients with acute kidney injury
Source: Crit Care. 2013 Oct 22;17(5):R250. doi: 10.1186/cc13076 (PMC4056803; doi:10.1186/cc13076)
Supplement: Additional file 2 — Comparison of characteristics of patients in this study and patients in the FINNAKI study. [file cc13076-S2.doc]

**Additional file 2.** Comparison of characteristics of patients in this study and patients in the FINNAKI study

|  | **Patients in this study**  **(N=1568)**  **N (%) or median [IQR]** | **FINNAKI study patients (N=2901)**  **N (%) or median  [IQR]** | **P** |
| --- | --- | --- | --- |
| Age (years) | 65 (53-74) | 64 (51-74) | 0.020 |
| Gender (male) | 1015 (64.7) | 1846 (63.6) | 0.243 |
| Baseline serum/plasma creatinine (µmol/l) | 78 (63-95) | 76 (61-93) | 0.025 |
| **Co-morbidity** | | | |
| Chronic obstructive pulmonary disease | 169 (10.8) | 264 (9.2) | 0.041 |
| Hypertension | 759 (48.4) | 1379 (47.8) | 0.354 |
| Arteriosclerosis | 236 (15.1) | 380 (13.2) | 0.042 |
| Diabetes | 359 (22.9) | 637 (22.0) | 0.255 |
| Systolic heart failure | 219 (14.0) | 335 (11.7) | 0.013 |
| Chronic kidney disease | 127 (8.1) | 189 (6.5) | 0.029 |
| **Admission type** | | | |
| Emergency | 1287 (82.1) | 2544 (88.6) | <0.001 |
| Surgical | 618 (39.4) | 1010 (34.8) | 0.001 |
| **Severity and outcome** | | | |
| SOFA (first 24h, points) | 7 (5-10) | 7 (5-9) | 0.069 |
| SAPS II score (points) | 36 (27-49) | 37 (28-50) | 0.218 |
| Acute kidney injury | 635 (40.5) | 1141 (39.3) | 0.233 |
| Stage 1 | 280 (17.9) | 499 (17.2) | 0.265 |
| Stage 2 | 119 (7.6) | 232 (8.0) | 0.732 |
| Stage 3 | 236 (15.1) | 410 (14.1) | 0.417 |
| RRT | 162 (10.3) | 272 (9.4) | 0.164 |
| Length of ICU stay | 2.8 (1.6-5.4) | 2.6 (1.2-5.0) | <0.001 |
| Length of hospital stay | 9 (5-17) | 8 (4-16) | 0.001 |
| 90-day mortality | 350 (22.3) | 678 (23.4) | 0.224 |
| six-month mortality | 378 (24.1) | 732 (25.2) | 0.214 |
