# Supplementary material for: Six-month survival and quality of life of intensive care patients with acute kidney injury
Source: Crit Care. 2013 Oct 22;17(5):R250. doi: 10.1186/cc13076 (PMC4056803; doi:10.1186/cc13076)
Supplement: Additional file 3 — EQ-5D index at six months stratified into groups based on hospital length-of stay. [file cc13076-S3.doc]

**Additional file 3.** EQ-5D index at six months stratified into groups based on hospital length-of stay. (P<0.001 between groups, data available for 958/959 patients)

| **Hospital LOS, days** | **N (%)** | **EQ-5D index, median (IQR)** |
| --- | --- | --- |
| 0 - 7 | 351 (36.6) | 0.735 (0.533 – 1.00) |
| 8 - 14 | 315 (32.9) | 0.691 (0.533 – 1.00) |
| over 15 | 292 (30.5) | 0.645 (0.475 – 0.788) |
